# 1,2-Bis[(3,6,9-trimethyl-3,12-ep­oxy-3,4,5,5a,6,7,8,8a,9,10,12,12a-dodeca­hydro­pyrano[4,3-*j*][1,2]benzodioxepin-4-yl)­oxy]ethane

**DOI:** 10.1107/S1600536812005089

**Published:** 2012-02-10

**Authors:** Liwei Jia, Zhengyu Yue, Dongying Lv, Po Gao

**Affiliations:** a School of Pharmacy, Heilongjiang University of Chinese Medicine, Harbin 150040, People’s Republic of China; b School of Chemistry and Materials Science, Heilongjiang University, Harbin 150080, People’s Republic of China; c Heilongjiang Environmental Monitoring Central Station, Harbin 150056, People’s Republic of China

## Abstract

The title compound, C_32_H_50_O_10_, prepared from a mixture of α- and β-dihydro­artemisinin, has two β-arteether moieties linked *via* an –OCH_2_CH_2_O– bridge, so that the mol­ecule is symmetric about the bridge. Each asymmetric unit contains a β-arteether moiety and an –OCH_2_ group, which is only one-half of the mol­ecule. The endo-peroxide bridges of the parent compounds have been retained in each half of the diol-bridged dimer. The rings exhibit chair and twist-boat conformations.

## Related literature

For related literature and structures, see: Brossi *et al.* (1988 ▶); Dominguez Gerpe *et al.* (1988 ▶); Flack & Bernardinelli (2000 ▶); Flippen-Anderson *et al.* (1989 ▶); Haynes *et al.* (2002 ▶); Luo *et al.* (1984 ▶); Paik *et al.* (2006 ▶); Qinghaosu Research Group (1980 ▶); Venugopalan *et al.* (1995 ▶); Woerdenbag *et al.* (1993 ▶); Yue *et al.* (2006 ▶). For the synthesis, see: Posner *et al.* (1997 ▶). For puckering parameters, see: Cremer & Pople (1975 ▶).
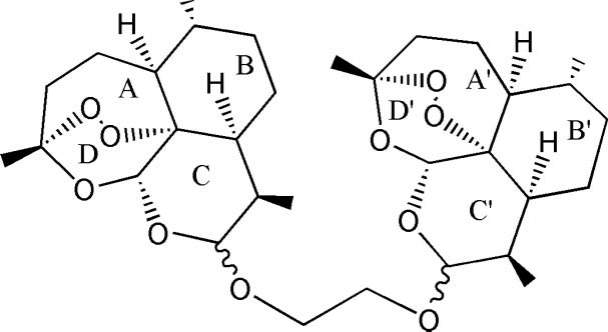


## Experimental

### 

#### Crystal data

C_32_H_50_O_10_
*M* *_r_* = 594.72
Monoclinic, 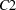

*a* = 18.033 (4) Å
*b* = 9.3127 (19) Å
*c* = 11.061 (2) Å
β = 123.58 (3)°
*V* = 1547.5 (8) Å^3^
*Z* = 2
Mo *K*α radiation
μ = 0.09 mm^−1^
*T* = 295 K
0.42 × 0.38 × 0.31 mm

#### Data collection

Rigaku R-AXIS RAPID diffractometer
Absorption correction: multi-scan (*ABSCOR*; Higashi, 1995 ▶) *T*_min_ = 0.962, *T*_max_ = 0.972
6717 measured reflections
1614 independent reflections
1195 reflections with *I* > 2σ(*I*)
*R*_int_ = 0.043

#### Refinement

*R*[*F* ^2^ > 2σ(*F* ^2^)] = 0.042
*wR*(*F* ^2^) = 0.129
*S* = 1.10
1614 reflections
194 parameters
1 restraint
H-atom parameters constrained
Δρ_max_ = 0.20 e Å^−3^
Δρ_min_ = −0.20 e Å^−3^

### 

Data collection: *RAPID-AUTO* (Rigaku, 1998 ▶); cell refinement: *RAPID-AUTO*; data reduction: *CrystalStructure* (Rigaku/MSC, 2002 ▶); program(s) used to solve structure: *SHELXS97* (Sheldrick, 2008 ▶); program(s) used to refine structure: *SHELXL97* (Sheldrick, 2008 ▶); molecular graphics: *ORTEPII* (Johnson, 1976 ▶); software used to prepare material for publication: *SHELXL97*.

## Supplementary Material

Crystal structure: contains datablock(s) I, global. DOI: 10.1107/S1600536812005089/jj2118sup1.cif

Structure factors: contains datablock(s) I. DOI: 10.1107/S1600536812005089/jj2118Isup2.hkl

Supplementary material file. DOI: 10.1107/S1600536812005089/jj2118Isup3.cml

Additional supplementary materials:  crystallographic information; 3D view; checkCIF report

## Figures and Tables

**Fig. 1.**
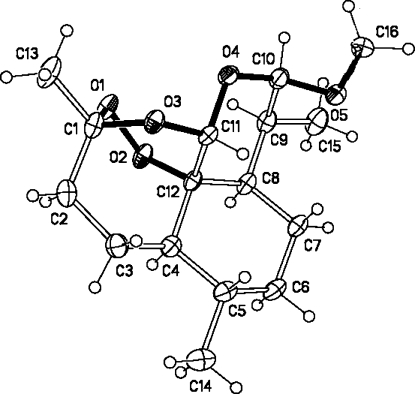
View of the asymmetric unit showing with the atom-labeling and with displacement ellipsoids drawn at the 30% probability level.

## supplementary crystallographic information

### Comment

Dihydroartemisinin is reported to be more therapeutically active than the parent compound. Some trioxane dimers have been found to possess high antimalarial activities (Venugopalan *et al.*, 1995) and moderate antitumor activities. (Woerdenbag *et al.*, 1993). The dihydroartemisinin triethylene glycol dimers, have strong *in vitro* growth-inhibitory activity. The dimer with β-stereochemistry at both of the lactol acetal positions is very active and highly antiproliferative (Posner *et al.*, 1997). We conclude, therefore, that the stereochemistry of the diol linkage is an important determinant for cytotoxicity. We chose the simplified analogue, the title compound, whose structure and activity have not been reported, to find out the relationship between the activity and stereo-structure. Hence, knowledge of the structure of the title compound is of interest and is reported here.

The X-ray structures of artemisinin and artemisinin derivatives have been reported, including dihydroartemisinin, artemether, artesunic acid (Luo *et al*., 1984), both *cis-*deoxyarteether (Brossi *et al.*, 1988) and *trans-*deoxyarteether (Dominguez Gerpe *et al.*, 1988), α-artesunate, β-artesunate (Haynes *et al.*, 2002), the symmetric form of the ether dimer of deoxydihydroartemisinin (Flippen-Anderson *et al.*,1989), the asymmetric form of the ether dimer of dihydroartemisinin (Yue *et al.*, 2006), and the phthalate dimer (Paik *et al.* 2006). Although the endoperoxide group is an important determinant for cytotoxicity, no crystal structure of a diol dimer of dihydroartemisinin with a peroxy unit has been reported previously. We report here the crystal structure of a diol dimer of dihydroartemisinin, the title compound, which is a diol dimer of dihydroartemisinin with a unique 1,2,4-trioxane peroxy bridge.

The title molecule is symmetrical. Each moiety of the dimer is totally the same, hence we describe only the asymmetric unit here.

The seven-membered ring *A*(C1/C2/C3/C4/C12/O2/O1) includes key peroxy linkages [O1—O2 = 1.463 (3) Å]. The length of peroxy linkages is very close to that of the ether dimer of dihydroartemisinin (1.467 (4) Å and 1.461 (3) Å respectively) (Yue *et al.*,2006). The six-membered ring *B*(C4/C5/C6/C7/C8/C12) has a distorted chair conformation, with Cremer & Pople (1975) puckering parameters Q, θ and φ of 0.5422 (42) Å, 8.33 (43)° and 150 (3)°. For an ideal chair, θ has a value of 0 or 180°. The six-membered ring *C*(O4/C10/C9/C8/C12/C11) also has a distorted chair conformation, with puckering parameters Q, θ and φ of 0.5157 (39) Å, 179.09 (44)°, 93 (20)°. Similar conformations were found in the corresponding six-membered rings of dihydroartemisinin(Luo *et al.*, 1984). The six-membered ring involving the endoperoxide bridges *D*(C1/O1/O2/C12/C11/O3), is best described by a twist-boat conformation, for which the puckering parameters Q, θ and φ are 0.7463 (38) Å, 85.77 (27)°, 335.6 (3)°. For an ideal twist-boat conformation, θ and φ are 90 and (60*n* + 30)° respectively. In contrast, the six-membered ring formed by the endoperoxide bridge in dihydroartemisinin has a distorted boat conformation.

### Experimental

The title compound has been prepared according to a literature procedure (Posner *et al.*, 1997). To a solution of dihydroartemisinin (297 mg, 1.05 mmol) in toluene (30 mL) at 293~298 K, glycol (0.029 mL, 0.53 mmol) was added followed by BF_3_Et_2_O (0.032 mL, 0.26 mmol). The reaction was stirred at the same temperature for 3 h. The mixture was then diluted with methylene chloride and was washed twice with water. The organic portions were collected, dried over (MgSO_4_) and concentrated. The crude product was purified by column chromatography (flash, 7–20% ethyl acetate/petro ether) to produce the title compound (50.7 mg, 0.085 mmol, yield 17%). Crystals were obtained from ether, diffused with hexane at room temperature.

### Refinement

The methyl H atoms were constrained to an ideal geometry (C—H = 0.96 Å) with *U*_iso_(H) = 1.5*U*_eq_(C), but were allowed to rotate freely about the C—C bonds. All other H atoms were placed in geometrically idealized positions and constrained to ride on their parent C atoms at distances of 0.97 or 0.98 Å for methylene or methine groups, respectively, and with *U*_iso_(H) = 1.2*U*_eq_(C). As there are no significant anomalous scatterers in the molecule, attempts to confirm the absolute structure by refinement of the Flack parameter (Flack & Bernardinelli, 2000) in the presence of 1614 sets of Friedel equivalents led to an inconclusive value for the parameter. Therefore, the Friedel pairs were merged before the final refinement and the absolute configuration was assigned to correspond to that determined for artemisinin (Qinghaosu Research Group, 1980).

### Crystal data

**Table:**

C_32_H_50_O_10_	*F*(000) = 644
*M**_r_* = 594.72	*D*_x_ = 1.276 Mg m^−^^3^
Monoclinic, *C*2	Mo *K*α radiation, λ = 0.71073 Å
Hall symbol: C 2y	Cell parameters from 5507 reflections
*a* = 18.033 (4) Å	θ = 3.7–26.0°
*b* = 9.3127 (19) Å	µ = 0.09 mm^−^^1^
*c* = 11.061 (2) Å	*T* = 295 K
β = 123.58 (3)°	Prism, colorless
*V* = 1547.5 (8) Å^3^	0.42 × 0.38 × 0.31 mm
*Z* = 2	

### Data collection

**Table:**

Rigaku R-AXIS RAPID diffractometer	1614 independent reflections
Radiation source: fine-focus sealed tube	1195 reflections with *I* > 2σ(*I*)
Graphite monochromator	*R*_int_ = 0.043
Detector resolution: 10.000 pixels mm^-1^	θ_max_ = 26.0°, θ_min_ = 3.7°
ω scans	*h* = −22→22
Absorption correction: multi-scan (*ABSCOR*; Higashi, 1995)	*k* = −11→9
*T*_min_ = 0.962, *T*_max_ = 0.972	*l* = −13→13
6717 measured reflections	

### Refinement

**Table:**

Refinement on *F*^2^	Primary atom site location: structure-invariant direct methods
Least-squares matrix: full	Secondary atom site location: difference Fourier map
*R*[*F*^2^ > 2σ(*F*^2^)] = 0.042	Hydrogen site location: inferred from neighbouring sites
*wR*(*F*^2^) = 0.129	H-atom parameters constrained
*S* = 1.10	*w* = 1/[σ^2^(*F*_o_^2^) + (0.0715*P*)^2^ + 0.1671*P*] where *P* = (*F*_o_^2^ + 2*F*_c_^2^)/3
1614 reflections	(Δ/σ)_max_ < 0.001
194 parameters	Δρ_max_ = 0.20 e Å^−^^3^
1 restraint	Δρ_min_ = −0.20 e Å^−^^3^

### Special details

**Table:**

Geometry. All e.s.d.'s (except the e.s.d. in the dihedral angle between two l.s. planes) are estimated using the full covariance matrix. The cell e.s.d.'s are taken into account individually in the estimation of e.s.d.'s in distances, angles and torsion angles; correlations between e.s.d.'s in cell parameters are only used when they are defined by crystal symmetry. An approximate (isotropic) treatment of cell e.s.d.'s is used for estimating e.s.d.'s involving l.s. planes.
Refinement. Refinement of *F*^2^ against ALL reflections. The weighted *R*-factor *wR* and goodness of fit *S* are based on *F*^2^, conventional *R*-factors *R* are based on *F*, with *F* set to zero for negative *F*^2^. The threshold expression of *F*^2^ > σ(*F*^2^) is used only for calculating *R*-factors(gt) *etc*. and is not relevant to the choice of reflections for refinement. *R*-factors based on *F*^2^ are statistically about twice as large as those based on *F*, and *R*- factors based on ALL data will be even larger.

### Fractional atomic coordinates and isotropic or equivalent isotropic displacement parameters (Å^2^)

**Table:**

	*x*	*y*	*z*	*U*_iso_*/*U*_eq_	
O1	0.85534 (15)	0.7920 (3)	0.4203 (3)	0.0696 (7)	
O2	0.81128 (13)	0.6711 (3)	0.4401 (2)	0.0638 (7)	
O3	0.76331 (14)	0.7918 (3)	0.1695 (2)	0.0578 (6)	
O4	0.68018 (14)	0.8818 (2)	0.2453 (3)	0.0600 (6)	
O5	0.52997 (15)	0.8430 (3)	0.1553 (3)	0.0655 (7)	
C1	0.8536 (2)	0.7701 (5)	0.2924 (4)	0.0638 (9)	
C2	0.8844 (2)	0.6212 (5)	0.2839 (5)	0.0741 (11)	
H2A	0.9172	0.6284	0.2382	0.089*	
H2B	0.9251	0.5858	0.3818	0.089*	
C3	0.8104 (2)	0.5128 (4)	0.2012 (5)	0.0694 (10)	
H3A	0.8369	0.4211	0.2041	0.083*	
H3B	0.7740	0.5427	0.1005	0.083*	
C4	0.7496 (2)	0.4904 (4)	0.2559 (4)	0.0590 (8)	
H4A	0.7836	0.4307	0.3431	0.071*	
C5	0.6668 (2)	0.4016 (4)	0.1475 (4)	0.0652 (9)	
H5	0.6336	0.4546	0.0557	0.078*	
C6	0.6066 (2)	0.3825 (4)	0.2020 (4)	0.0700 (10)	
H6A	0.5535	0.3308	0.1298	0.084*	
H6B	0.6371	0.3255	0.2901	0.084*	
C7	0.5804 (2)	0.5246 (4)	0.2323 (4)	0.0642 (9)	
H7A	0.5408	0.5085	0.2645	0.077*	
H7B	0.5485	0.5805	0.1435	0.077*	
C8	0.6620 (2)	0.6093 (4)	0.3487 (4)	0.0576 (8)	
H8	0.6917	0.5495	0.4362	0.069*	
C9	0.6407 (2)	0.7511 (4)	0.3906 (4)	0.0634 (9)	
H9	0.6964	0.7825	0.4786	0.076*	
C10	0.6163 (2)	0.8677 (4)	0.2800 (4)	0.0643 (9)	
H10	0.6147	0.9586	0.3231	0.077*	
C11	0.6992 (2)	0.7533 (3)	0.1973 (4)	0.0505 (7)	
H11	0.6452	0.7232	0.1059	0.061*	
C12	0.72923 (18)	0.6310 (4)	0.3060 (3)	0.0497 (7)	
C13	0.9078 (3)	0.8911 (6)	0.2898 (5)	0.0916 (15)	
H13A	0.8908	0.9796	0.3125	0.137*	
H13B	0.8974	0.8977	0.1950	0.137*	
H13C	0.9698	0.8732	0.3603	0.137*	
C14	0.6926 (4)	0.2560 (5)	0.1183 (6)	0.0950 (14)	
H14A	0.7275	0.2040	0.2078	0.143*	
H14B	0.7269	0.2700	0.0768	0.143*	
H14C	0.6398	0.2025	0.0520	0.143*	
C15	0.5731 (3)	0.7405 (6)	0.4325 (5)	0.0923 (15)	
H15A	0.5162	0.7137	0.3490	0.138*	
H15B	0.5684	0.8318	0.4680	0.138*	
H15C	0.5924	0.6693	0.5070	0.138*	
C16	0.4917 (3)	0.9620 (4)	0.0595 (5)	0.0737 (10)	
H16A	0.5156	1.0498	0.1152	0.088*	
H16B	0.4280	0.9619	0.0162	0.088*	

### Atomic displacement parameters (Å^2^)

**Table:**

	*U*^11^	*U*^22^	*U*^33^	*U*^12^	*U*^13^	*U*^23^
O1	0.0611 (13)	0.0951 (18)	0.0531 (14)	−0.0343 (13)	0.0319 (11)	−0.0154 (14)
O2	0.0523 (11)	0.0890 (17)	0.0424 (12)	−0.0224 (12)	0.0214 (9)	−0.0031 (12)
O3	0.0544 (11)	0.0700 (15)	0.0532 (13)	−0.0115 (10)	0.0324 (10)	0.0023 (11)
O4	0.0605 (13)	0.0547 (13)	0.0710 (16)	−0.0134 (11)	0.0401 (12)	−0.0100 (12)
O5	0.0583 (12)	0.0607 (14)	0.0762 (17)	−0.0088 (11)	0.0364 (12)	−0.0090 (13)
C1	0.0488 (17)	0.091 (2)	0.0528 (19)	−0.0159 (17)	0.0287 (15)	−0.0025 (19)
C2	0.0547 (19)	0.098 (3)	0.073 (3)	0.002 (2)	0.0372 (18)	0.007 (2)
C3	0.067 (2)	0.078 (3)	0.067 (2)	0.0031 (18)	0.0399 (19)	0.002 (2)
C4	0.0566 (17)	0.061 (2)	0.050 (2)	−0.0021 (15)	0.0237 (15)	0.0081 (16)
C5	0.073 (2)	0.056 (2)	0.055 (2)	−0.0097 (17)	0.0284 (18)	0.0009 (17)
C6	0.073 (2)	0.060 (2)	0.062 (2)	−0.0256 (18)	0.0282 (18)	−0.0002 (19)
C7	0.0589 (18)	0.068 (2)	0.065 (2)	−0.0213 (16)	0.0337 (17)	−0.0048 (18)
C8	0.0548 (17)	0.071 (2)	0.0455 (18)	−0.0178 (15)	0.0267 (15)	−0.0040 (17)
C9	0.0580 (18)	0.086 (2)	0.054 (2)	−0.0178 (17)	0.0354 (16)	−0.0146 (19)
C10	0.0571 (17)	0.068 (2)	0.074 (2)	−0.0143 (17)	0.0399 (17)	−0.020 (2)
C11	0.0509 (17)	0.0509 (17)	0.0500 (18)	−0.0146 (13)	0.0281 (15)	−0.0052 (14)
C12	0.0446 (14)	0.0619 (19)	0.0361 (15)	−0.0144 (13)	0.0183 (12)	−0.0016 (14)
C13	0.073 (2)	0.129 (4)	0.079 (3)	−0.044 (3)	0.046 (2)	−0.013 (3)
C14	0.116 (3)	0.066 (3)	0.100 (4)	−0.009 (2)	0.057 (3)	−0.013 (3)
C15	0.087 (3)	0.129 (4)	0.086 (3)	−0.030 (3)	0.063 (3)	−0.024 (3)
C16	0.071 (2)	0.0533 (19)	0.093 (3)	0.0042 (17)	0.043 (2)	−0.005 (2)

### Geometric parameters (Å, º)

**Table:**

O1—C1	1.412 (4)	C6—H6B	0.9700
O1—O2	1.463 (3)	C7—C8	1.532 (4)
O2—C12	1.450 (3)	C7—H7A	0.9700
O3—C11	1.397 (3)	C7—H7B	0.9700
O3—C1	1.446 (4)	C8—C9	1.518 (5)
O4—C10	1.407 (4)	C8—C12	1.538 (4)
O4—C11	1.426 (4)	C8—H8	0.9800
O5—C10	1.416 (4)	C9—C10	1.508 (5)
O5—C16	1.421 (5)	C9—C15	1.528 (5)
C1—C13	1.502 (5)	C9—H9	0.9800
C1—C2	1.516 (6)	C10—H10	0.9800
C2—C3	1.512 (6)	C11—C12	1.522 (4)
C2—H2A	0.9700	C11—H11	0.9800
C2—H2B	0.9700	C13—H13A	0.9600
C3—C4	1.532 (5)	C13—H13B	0.9600
C3—H3A	0.9700	C13—H13C	0.9600
C3—H3B	0.9700	C14—H14A	0.9600
C4—C5	1.539 (5)	C14—H14B	0.9600
C4—C12	1.544 (5)	C14—H14C	0.9600
C4—H4A	0.9800	C15—H15A	0.9600
C5—C6	1.515 (5)	C15—H15B	0.9600
C5—C14	1.525 (6)	C15—H15C	0.9600
C5—H5	0.9800	C16—C16^i^	1.504 (9)
C6—C7	1.504 (5)	C16—H16A	0.9700
C6—H6A	0.9700	C16—H16B	0.9700
			
C1—O1—O2	109.0 (2)	C7—C8—H8	106.5
C12—O2—O1	112.1 (2)	C12—C8—H8	106.5
C11—O3—C1	113.3 (2)	C10—C9—C8	112.8 (3)
C10—O4—C11	115.2 (2)	C10—C9—C15	111.6 (3)
C10—O5—C16	114.8 (3)	C8—C9—C15	114.6 (3)
O1—C1—O3	108.1 (3)	C10—C9—H9	105.6
O1—C1—C13	104.9 (3)	C8—C9—H9	105.6
O3—C1—C13	106.5 (3)	C15—C9—H9	105.6
O1—C1—C2	112.8 (3)	O4—C10—O5	112.0 (3)
O3—C1—C2	109.3 (3)	O4—C10—C9	111.9 (3)
C13—C1—C2	114.8 (3)	O5—C10—C9	110.1 (3)
C3—C2—C1	114.7 (3)	O4—C10—H10	107.6
C3—C2—H2A	108.6	O5—C10—H10	107.6
C1—C2—H2A	108.6	C9—C10—H10	107.6
C3—C2—H2B	108.6	O3—C11—O4	105.4 (2)
C1—C2—H2B	108.6	O3—C11—C12	113.0 (2)
H2A—C2—H2B	107.6	O4—C11—C12	112.8 (2)
C2—C3—C4	115.9 (3)	O3—C11—H11	108.5
C2—C3—H3A	108.3	O4—C11—H11	108.5
C4—C3—H3A	108.3	C12—C11—H11	108.5
C2—C3—H3B	108.3	O2—C12—C11	109.4 (2)
C4—C3—H3B	108.3	O2—C12—C8	104.6 (2)
H3A—C3—H3B	107.4	C11—C12—C8	110.1 (2)
C3—C4—C5	111.3 (3)	O2—C12—C4	106.0 (2)
C3—C4—C12	113.0 (3)	C11—C12—C4	113.7 (2)
C5—C4—C12	114.5 (3)	C8—C12—C4	112.5 (2)
C3—C4—H4A	105.7	C1—C13—H13A	109.5
C5—C4—H4A	105.7	C1—C13—H13B	109.5
C12—C4—H4A	105.7	H13A—C13—H13B	109.5
C6—C5—C14	110.4 (3)	C1—C13—H13C	109.5
C6—C5—C4	110.7 (3)	H13A—C13—H13C	109.5
C14—C5—C4	111.3 (3)	H13B—C13—H13C	109.5
C6—C5—H5	108.1	C5—C14—H14A	109.5
C14—C5—H5	108.1	C5—C14—H14B	109.5
C4—C5—H5	108.1	H14A—C14—H14B	109.5
C7—C6—C5	111.5 (3)	C5—C14—H14C	109.5
C7—C6—H6A	109.3	H14A—C14—H14C	109.5
C5—C6—H6A	109.3	H14B—C14—H14C	109.5
C7—C6—H6B	109.3	C9—C15—H15A	109.5
C5—C6—H6B	109.3	C9—C15—H15B	109.5
H6A—C6—H6B	108.0	H15A—C15—H15B	109.5
C6—C7—C8	111.5 (3)	C9—C15—H15C	109.5
C6—C7—H7A	109.3	H15A—C15—H15C	109.5
C8—C7—H7A	109.3	H15B—C15—H15C	109.5
C6—C7—H7B	109.3	O5—C16—C16^i^	113.8 (3)
C8—C7—H7B	109.3	O5—C16—H16A	108.8
H7A—C7—H7B	108.0	C16^i^—C16—H16A	108.8
C9—C8—C7	114.6 (3)	O5—C16—H16B	108.8
C9—C8—C12	110.8 (3)	C16^i^—C16—H16B	108.8
C7—C8—C12	111.3 (3)	H16A—C16—H16B	107.7
C9—C8—H8	106.5		
			
C1—O1—O2—C12	−44.0 (3)	C8—C9—C10—O4	−51.6 (4)
O2—O1—C1—O3	72.2 (3)	C15—C9—C10—O4	177.6 (3)
O2—O1—C1—C13	−174.5 (3)	C8—C9—C10—O5	73.6 (3)
O2—O1—C1—C2	−48.8 (3)	C15—C9—C10—O5	−57.2 (4)
C11—O3—C1—O1	−31.6 (4)	C1—O3—C11—O4	92.6 (3)
C11—O3—C1—C13	−143.8 (3)	C1—O3—C11—C12	−31.0 (4)
C11—O3—C1—C2	91.6 (3)	C10—O4—C11—O3	−180.0 (3)
O1—C1—C2—C3	94.4 (4)	C10—O4—C11—C12	−56.3 (3)
O3—C1—C2—C3	−25.9 (4)	O1—O2—C12—C11	−17.4 (3)
C13—C1—C2—C3	−145.5 (4)	O1—O2—C12—C8	−135.4 (2)
C1—C2—C3—C4	−56.7 (5)	O1—O2—C12—C4	105.6 (3)
C2—C3—C4—C5	168.8 (3)	O3—C11—C12—O2	57.2 (3)
C2—C3—C4—C12	38.4 (4)	O4—C11—C12—O2	−62.2 (3)
C3—C4—C5—C6	−179.1 (3)	O3—C11—C12—C8	171.6 (3)
C12—C4—C5—C6	−49.4 (4)	O4—C11—C12—C8	52.2 (3)
C3—C4—C5—C14	57.7 (4)	O3—C11—C12—C4	−61.0 (3)
C12—C4—C5—C14	−172.7 (3)	O4—C11—C12—C4	179.6 (2)
C14—C5—C6—C7	179.8 (3)	C9—C8—C12—O2	68.0 (3)
C4—C5—C6—C7	56.0 (4)	C7—C8—C12—O2	−163.2 (3)
C5—C6—C7—C8	−60.4 (4)	C9—C8—C12—C11	−49.4 (3)
C6—C7—C8—C9	−177.2 (3)	C7—C8—C12—C11	79.4 (3)
C6—C7—C8—C12	56.1 (4)	C9—C8—C12—C4	−177.4 (3)
C7—C8—C9—C10	−77.1 (3)	C7—C8—C12—C4	−48.6 (4)
C12—C8—C9—C10	49.9 (4)	C3—C4—C12—O2	−71.1 (3)
C7—C8—C9—C15	52.1 (4)	C5—C4—C12—O2	160.1 (3)
C12—C8—C9—C15	179.1 (3)	C3—C4—C12—C11	49.1 (4)
C11—O4—C10—O5	−69.3 (4)	C5—C4—C12—C11	−79.8 (3)
C11—O4—C10—C9	54.9 (4)	C3—C4—C12—C8	175.2 (3)
C16—O5—C10—O4	−69.2 (4)	C5—C4—C12—C8	46.4 (4)
C16—O5—C10—C9	165.7 (3)	C10—O5—C16—C16^i^	92.8 (4)

Symmetry code: (i) −*x*+1, *y*, −*z*.
